# The NLRP3 Inflammasome: An Overview of Mechanisms of Activation and Regulation

**DOI:** 10.3390/ijms20133328

**Published:** 2019-07-06

**Authors:** Nathan Kelley, Devon Jeltema, Yanhui Duan, Yuan He

**Affiliations:** Department of Biochemistry, Microbiology and Immunology, Wayne State University School of Medicine, Detroit, MI 48201, USA

**Keywords:** NLRP3 inflammasome, Priming, Ionic flux, ROS, Mitochondrial dysfunction, Lysosomal damage, Post-translational modification, NLRP3 regulators

## Abstract

The NLRP3 inflammasome is a critical component of the innate immune system that mediates caspase-1 activation and the secretion of proinflammatory cytokines IL-1β/IL-18 in response to microbial infection and cellular damage. However, the aberrant activation of the NLRP3 inflammasome has been linked with several inflammatory disorders, which include cryopyrin-associated periodic syndromes, Alzheimer’s disease, diabetes, and atherosclerosis. The NLRP3 inflammasome is activated by diverse stimuli, and multiple molecular and cellular events, including ionic flux, mitochondrial dysfunction, and the production of reactive oxygen species, and lysosomal damage have been shown to trigger its activation. How NLRP3 responds to those signaling events and initiates the assembly of the NLRP3 inflammasome is not fully understood. In this review, we summarize our current understanding of the mechanisms of NLRP3 inflammasome activation by multiple signaling events, and its regulation by post-translational modifications and interacting partners of NLRP3.

## 1. Introduction

The innate immune system is the first line of host defense and the engagement of germline-encoded pattern-recognition receptors (PRRs) activate it in response to harmful stimuli, such as invading pathogens, dead cells, or environmental irritants [1]. PRRs recognize the presence of unique microbial components, called pathogen-associated molecular patterns (PAMPs) or damage-associated molecular patterns (DAMPs), which are generated by endogenous stress, and trigger downstream inflammatory pathways to eliminate microbial infection and repair damaged tissues. The activation of inflammasomes, a group of intracellular multimeric protein complexes that activate inflammatory caspase-1, is a major inflammatory pathway [2]. An inflammasome is defined by its sensor protein (a PRR), which oligomerizes to form a pro-caspase-1 activating platform in response to DAMPs or PAMPs. There are five members of PRRs that have been confirmed to form inflammasomes: the nucleotide-binding oligomerization domain (NOD), leucine-rich repeat (LRR)-containing proteins (NLR) family members NLRP1, NLRP3, and NLRC4, as well as absent-in-melanoma 2 (AIM2) and pyrin [3,4]. In addition, other members of PRRs, such as NLRP2, NLRP6, NLRP7, NLRP12, and IFI16, were also reported to form inflammasomes [5,6,7,8,9]. In the case of some of these PRRs, such as NLRP1, NLRP3, AIM2, and pyrin, a bipartite adaptor protein, which is known as apoptosis-associated speck-like protein containing a caspase-recruitment domain (ASC), facilitates the recruitment of pro-caspase-1 to the inflammasome complex [10].

Caspase-1 is activated via proximity-induced autocatalytic activation upon recruitment to an inflammasome. Active caspase-1 cleaves the cytokines pro-interleukin-1β (pro-IL-1β) and pro-IL-18 into their mature and biologically active forms [11,12,13]. IL-1β induces the expression of genes that control fever, pain threshold, vasodilatation, and hypotension, and its reception leads to an endothelial cell response that facilitates the infiltration of immune cells to infected or damaged tissues [14]. IL-18 is necessary for interferon-gamma (IFN-γ) production and it is a co-stimulatory cytokine that mediates adaptive immunity [14]. Active caspase-1 also cleaves gasdermin D (GSDMD), which allows the N-terminal domain of GSDMD to form pores in the plasma membrane, thereby triggering a lytic, pro-inflammatory form of cell death, termed pyroptosis [15,16,17,18]. Pyroptosis forces intracellular pathogens out of their replicative niche, exposing them to other immune factors, and it also triggers cytokine release and the generation of DAMPs to further prepare the immune system to respond to infection [18,19].

The NLRP3 inflammasome is critical for host immune defenses against bacterial, fungal, and viral infections [20,21,22,23]; however, it has been linked to the pathogenesis of several inflammatory disorders when dysregulated, including cryopyrin-associated periodic syndromes (CAPS), Alzheimer’s disease, diabetes, gout, autoinflammatory diseases, and atherosclerosis [24,25]. NLRP3 is a tripartite protein that consists of an amino-terminal pyrin domain (PYD), a central nucleotide-binding and oligomerization domain (NOD; a.k.a. the NACHT domain), and a C-terminal leucine-rich repeat (LRR) domain [12]. The pyrin domain of NLRP3 interacts with the pyrin domain of ASC to initiate inflammasome assembly [26]. The NOD domain has ATPase activity that is required for NLRP3 oligomerization following activation [27]. This ATPase activity has recently been implicated as the target of MCC950, which is a commonly used NLRP3 inhibitor [28,29,30]. A recent study shows that the LRR domain is not involved in the autoinhibition of NLRP3, as previously suspected, and it is dispensable for NLRP3 inflammasome activation [31]. Therefore, the function of the LRR domain remains unclear.

**Figure 1:**
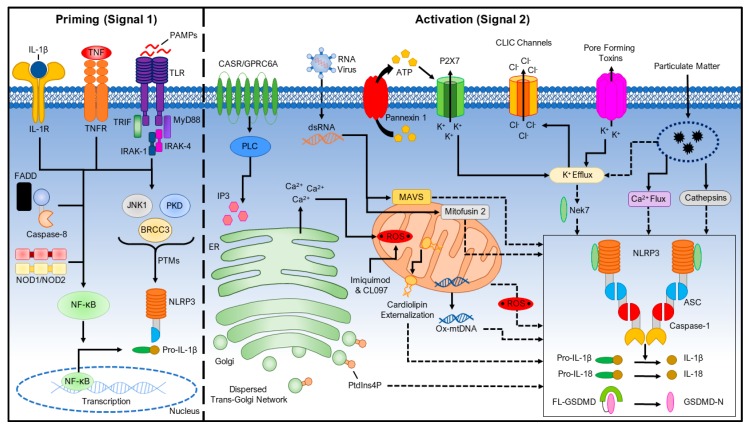
A Two-Signal Model for NLRP3 Inflammasome Activation. The priming signal (signal 1, **left**) is provided by microbial components or endogenous cytokines, leading to the activation of the transcription factor NF-κB and subsequent upregulation of NLRP3 and pro-interleukin-1β (pro-IL-1β). Caspase-8 and FAS-mediated death domain protein (FADD), and NOD1/2 are involved in the priming step by regulating the NF-κB pathway. NLRP3 undergoes post-translational modifications that license its activation. The activation signal (signal 2, **right**) is provided by a variety of stimuli including extracellular ATP, pore-forming toxins, RNA viruses, and particulate matter. Multiple molecular or cellular events, including ionic flux, mitochondrial dysfunction and reactive oxygen species (ROS) generation, and lysosomal damage, have been shown to activate the NLRP3 inflammasome. BRCC3, BRCA1/BRCA2-containing complex subunit 3; IL-1R, IL-1β receptor; JNK1, JUN N-terminal kinase 1; PKD, protein kinase D; TLR, toll-like receptor; TNFR, tumor necrosis factor receptor.

It is unlikely that NLRP3 directly binds to those stimuli since the NLRP3 inflammasome is activated by a wide range of stimuli that are structurally and chemically dissimilar [32]. Instead, it is suggested that NLPR3 senses a common cellular event induced by its stimuli. However, the identity of this cellular event remains controversial in the field. Currently, a two-signal model has been proposed for NLRP3 inflammasome activation (Figure 1).

In this model, a first signal that is provided by microbial components or endogenous cytokines primes the NLRP3 inflammasome; a second signal from extracellular ATP, pore-forming toxins, or particulate matter activates the NLRP3 inflammasome. Furthermore, multiple post-translational modifications and interacting partners of NLRP3 have been identified for regulating NLRP3 inflammasome activation. In this review, we focus on our current understanding of the mechanisms of NLRP3 inflammasome activation, and its regulation by post-translational modifications and interacting partners of NLRP3.

## 2. Priming the NLRP3 Inflammasome (Signal 1)

For macrophages, the presence of NLRP3 activators alone is insufficient for inducing inflammasome activation and a priming signal (signal 1) is required for its activation [33]. Macrophages must first be exposed to priming stimuli, such as ligands for toll-like receptors (TLRs), NLRs (e.g. NOD1 and NOD2), or cytokine receptors, which activate the transcription factor NF-κB. NF-κB upregulates the expression of NLRP3, which is thought to exist at concentrations that are inadequate for initiating inflammasome activation under resting conditions, and pro-IL-1β, which is not constitutively expressed in resting macrophages [33,34]. In contrast, priming signals do not appear to affect the expression levels of ASC, pro-caspase-1, and pro-IL-18 [33]. Moreover, both signaling molecules MyD88 and TRIF of the NF-κB signaling pathway regulate the induction of NLRP3 and pro-IL-1β in response to TLR ligands [33]. Recent studies indicate that both apoptotic signaling molecules caspase-8 and FADD are also required for the induction of NLRP3 during the priming process, and they do so independent of their apoptotic functions [35,36]. Caspase-8 interacts with the IKK complex, which is an essential element to NF-κB activation, following priming to promote its induction of NF-κB transcription and translocation [37]. FADD has a dual role in the NF-κB signaling pathway, as it is also reported to repress NF-κB activation by promoting apoptosis [38]. As the priming step is dispensable for NLRP3 inflammasome activation in *Nlrp3^-/-^* macrophages that are stably reconstituted with NLRP3 by viral transfection, the priming signal is thought to mainly play a transcriptional role in NLRP3 inflammasome activation by up-regulating NLRP3 and pro-IL-1β [33].

However, transcription-independent roles of the priming signal have been recently appreciated. In a rapid priming procedure, acute priming with lipopolysaccharides (LPS) (10 min. stimulation) enhances NLRP3 inflammasome activation in the absence of NLRP3 induction [39,40]. IL-1 receptor-associated kinase 1 (IRAK-1), which is a signaling molecule downstream of TLRs and MyD88, mediates this rapid transcription-independent priming [41,42]. LPS-induced phosphorylation of IRAK-1 promotes inflammasome activation in a manner that is independent of the IKK complex, which suggests that downstream NF-κB signaling is not essential to IRAK-1’s role in promoting inflammasome activation [43]. While the upregulation of NLRP3 will result in a more robust inflammatory response, these reports show that it is not necessary for inflammasome activation, which suggests that the priming step does more to license NLRP3 inflammasome activation beyond the transcriptional level. Furthermore, NLRP3 is ubiquitinated in its LRR domain, and priming signals induce its deubiquitination through the deubiquitinating enzyme BRCC3 (BRCC36 in humans), which is a JAMM domain-containing Zn^2+^ metalloprotease, to promote NLRP3 inflammasome activation [39,44,45]. Priming signals also trigger JNK1-mediated NLRP3 phosphorylation, which is a critical event for NLRP3 self-association and inflammasome activation [46]. Additionally, a recent study shows that priming signals downstream of TLR4 activate transcriptional factor IRF1 to induce the synthesis of mitochondrial DNA (mtDNA), which is required for NLRP3 inflammasome activation [47]. In conclusion, the priming signals regulate NLRP3 inflammasome activation through both transcription-dependent and -independent pathways (Figure 1).

## 3. Activating the NLRP3 Inflammasome (Signal 2)

NLRP3 can be activated by a wide range of stimuli following this priming step, including ATP, K^+^ ionophores [48], heme [49,50], particulate matter [51,52,53], pathogen-associated RNA [54,55,56,57], and bacterial and fungal toxins and components [58,59,60,61,62,63]. NLRP3 has not been observed to directly interact with any of these agonists and, due to their biochemical dissimilarity, it is suspected that they induce a common cellular signal. Currently, multiple molecular and cellular signaling events that are induced by NLRP3 stimuli, including ionic flux, mitochondrial dysfunction and the production of reactive oxygen species (ROS), and lysosomal damage, have been shown to activate the NLRP3 inflammasome (Figure 1).

### 3.1. Ionic Flux

NLRP3 stimuli induce ionic flux events in treated cells, including K^+^ efflux, Ca^2+^ mobilization, Cl^−^ efflux, and Na^+^ influx, which are implicated in activating the NLRP3 inflammasome.

#### 3.1.1. K^+^ Efflux

K^+^ efflux has long been recognized as a common ionic event that occurs in cells treated with most NLRP3 stimuli. Cytosolic depletion of K^+^ was found to mediate IL-1β maturation and release from macrophages and monocytes in response to ATP or nigericin in studies antecedent the discovery of inflammasomes, which are now known to be NLRP3 stimuli [48,64,65,66]. Furthermore, K^+^ efflux alone is capable of activating NLRP3, and high extracellular K^+^ blocks the activation of the NLRP3 inflammasome, but not the NLRC4 or AIM2 inflammasome [67,68]. Therefore, a decrease of intracellular K^+^ has been considered to be the common trigger for NLRP3 inflammasome activation [67]. Moreover, K^+^ efflux is also required for NLRP3 activation in the caspase-11-mediated non-canonical inflammasome pathway, but it is dispensable for the alternative NLRP3 inflammasome pathway [69,70,71]. Potassium efflux also leads to the activation of Ca^2+^ independent phospholipase A2, which promotes IL-1β maturation [65].

However, recent studies have identified some small chemical compounds, such as GB111-NH_2_, imiquimod, and CL097, which are able to activate NLRP3 independently of potassium efflux, which suggests that, either, there is an event further downstream of K^+^ efflux that causes NLRP3 inflammasome activation, or that K^+^ efflux-independent pathways exist for triggering NLRP3 inflammasome activation [72,73]. Additionally, mouse macrophages harboring a mutant NLRP3 (NLRP3^R258W^), which corresponded to the mutation in Muckle–Wells syndrome, activate the inflammasome following only LPS stimulation, and it occurs in the absence of K^+^ efflux [67,74]. Collectively, these findings suggest that K^+^ efflux is sufficient, but not necessary, for NLRP3 inflammasome activation. These findings also suggest that a drop of intracellular K^+^ may cause conformational changes of NLRP3 that can be induced by those small compounds, NLRP3-activating mutations, or signals from the alternative inflammasome pathway. Future studies are required for determining how NLRP3 senses changes in intracellular potassium concentration, or whether other proteins regulate its activation in response to K^+^ efflux.

#### 3.1.2. Ca^2+^ Mobilization

The role of Ca^2+^ mobilization in NLRP3 inflammasome activation remains controversial despite being prevalent in many intracellular signaling pathways [75]. Several earlier studies showed that the Ca^2+^ chelator BAPTA-AM inhibits IL-1β secretion, which suggests the involvement of Ca^2+^ mobilization in NLRP3 inflammasome activation [76,77,78]. Moreover, it was shown that multiple NLRP3 stimuli, such as ATP, nigericin, and particulate matter, induce changes in intracellular Ca^2+^ concentration [79]. Multiple sources of Ca^2+^ were reported to contribute to the increase of intracellular Ca^2+^ during the process of NLRP3 inflammasome activation. Phospholipase C is activated downstream of G-protein-coupled receptors (GPCRs) upon stimulation by NLRP3 stimuli, such as the calcium-sensing receptor (CaSR) and GPRC6A, and then hydrolyzes phosphatidylinositol 4,5-bisphosphate (PIP2) to produce inositol 1,4,5-triphosphate (IP3) [79]. IP3 then induces Ca^2+^ efflux from the lumen of the endoplasmic reticulum (ER) to the cytosol through a ligand-gated ion channel, which is called the IP3 receptor (IP3R) [79]. Furthermore, the inhibition of IP3R by 2-aminoethoxy diphenylborinate (2APB) prevents Ca^2+^ flux and NLRP3 activation [80]. However, 2APB was later found to inhibit NLRP3 activation independent of its ability to inhibit calcium mobilization, and it has since been used as a basis for designing NLRP3 inhibitors [81,82]. Additionally, the increase of Ca^2+^ in the cytosol can also result from the entry of Ca^2+^ through plasma membrane-resident Ca^2+^ channels, such as P2RX7, TRPM2, TRPM7, and TRPV2 [79,83,84]. The lysosome is also considered as a source of Ca^2+^ that may contribute to NLRP3 inflammasome activation [85]. Regardless of source, this stimuli-induced increase of cytosolic Ca^2+^ was shown to be critical for NLRP3 inflammasome activation, as the inhibition of the ER or plasma membrane Ca^2+^ channels attenuates caspase-1 activation and IL-1β secretion in response to NLRP3 stimuli.

However, how an increase in cytosolic Ca^2+^ promotes NLRP3 inflammasome activation is not yet clear. One study suggests that Ca^2+^ directly regulates NLRP3 inflammasome activation, as an increase of Ca^2+^ can promote interaction between NLRP3 and ASC in cell lysates of macrophages [80]. Alternatively, it is proposed that the increase of cytosolic Ca^2+^ causes Ca^2+^ overloading of mitochondria, which induces mitochondrial dysfunction that leads to NLRP3 inflammasome activation [79]. In contrast to those findings, another study shows that NLRP3 inflammasome activation that is induced by certain stimuli, such as ATP, nigericin, and lysosomotropic peptide Leu-Leu-OMe, is independent of Ca^2+^ mobilization, and that BAPTA-AM inhibits inflammasome activation independently of its ability to sequester Ca^2+^ [81]. Furthermore, K^+^ efflux-inducing stimuli can trigger NLRP3 inflammasome activation in macrophages that were incubated with Ca^2+^-free medium, which suggests that at least the extracellular pool of Ca^2+^ is not required for NLRP3 inflammasome activation [76,81]. In conclusion, Ca^2+^ mobilization might not be essential for NLRP3 inflammasome activation, though it may play a regulatory role in NLRP3 inflammasome activation under certain conditions.

#### 3.1.3. Na^+^ Influx and Cl^−^ Efflux

Na^+^ influx and Cl^−^ efflux are two additional ionic events that are implicated in NLRP3 inflammasome activation. Blocking the Na^+^ influx by reducing extracellular Na^+^ inhibits NLRP3 inflammasome activation that is induced by K^+^-free medium, gramicidin, or nigericin, but not by ATP, aerolysin, Al(OH)_3_, or silica [67]. Decreased Na^+^ influx was found to increase the threshold of K^+^ efflux for NLRP3 activation by low-K^+^ medium. Additionally, it was reported that monosodium urate crystals (MSU), upon delivery to lysosomes, cause an increase in intracellular Na^+^, leading to water influx and cellular swelling, which in turn lowers the intracellular K^+^ concentration [86]. In this way, Na^+^ may reduce the K^+^ concentration below the 90 mM threshold to induce NLRP3 inflammasome activation. However, Na^+^ influx alone induced by Na^+^ ionophores does not activate the NLRP3 inflammasome [67]. Therefore, Na^+^ influx plays a regulatory role in NLRP3 inflammasome activation, possibly by modulating the K^+^ efflux induced by stimuli.

The role of Cl^−^ influx in NLRP3 inflammasome activation was first suggested in one study, showing that a decrease in extracellular Cl^−^ concentration (from 130 mM to 9 mM) enhances ATP-induced IL-1β maturation and secretion [87]. Conversely, the increase of extracellular Cl^−^ inhibits IL-1β secretion [87,88]. Subsequently, several studies found that Cl^−^ channel inhibitors, including 4,40-diisothiocyano-2,20-stilbenedisulfonic acid, 5-nitro-2-(3-phenylpropylamino) benzoic acid, flufenamic acid, mefenamic acid, and indanyloxyacetic acid 94, can block the activation of the NLRP3 inflammasome, but not the NLRC4 or AIM2 inflammasome [83,89,90]. Chloride channels, which include the volume-regulated anion channel (VRAC) and chloride intracellular channels (CLICs), are reported to regulate NLRP3 inflammasome activation [83,89,91]. CLICs are translocated to the plasma membrane and trigger Cl^−^ efflux as a downstream event of mitochondrial dysfunction, which regulates NLRP3 inflammasome activation by promoting NLRP3-Nek7 interaction [89]. However, how Cl^−^ efflux promotes NLRP3-Nek7 interaction remains unknown. Interestingly, the knockdown of CLICs reduces pro-IL-1β mRNA expression and ASC-speck formation, but not NLRP3 protein levels [91]. It was also reported that Cl^−^ efflux can induce ASC-speck formation, but it does not lead to NLRP3 inflammasome activation without K^+^ efflux [92]. Further studies are warranted to define how Cl^−^ efflux coordinates with other ionic events to trigger NLRP3 inflammasome activation.

### 3.2. Reactive Oxygen Species (ROS) and Mitochondrial Dysfunction

The role of ROS and the mitochondria in NLRP3 inflammasome activation remains controversial. ROS was proposed as the common signal for NLRP3 inflammasome activation since most NLRP3 stimuli can induce ROS in treated cells, and the lysosomal NADPH oxidase was initially thought to be the source of ROS production [53,93]. However, several studies found that NLRP3 inflammasome activation is not affected in both mouse and human cells by either the genetic or pharmacological inhibition of NADPH oxidase [52,84,94]. More recently, a study shows that the deletion of superoxide generating NADPH oxidase 2 (NOX2) reduces the expression of NLRP3 in a traumatic brain injury model, and it disturbs the NLRP3-TXNIP interaction in the cerebral cortex of mice after ischemic stroke, but not in the umbilical vein endothelium, which suggests a tissue-specific role of cytosolic ROS in NLRP3 inflammasome activation [95]. Furthermore, NADPH oxidase 4 (NOX4) was shown to regulate carnitine palmitoyltransferase 1A (CPT1A) and cause increased fatty acid oxidation, which contributes to NLRP3 inflammasome activation [96].

Mitochondria are another source that are known to produce ROS through their respiratory functions and they are reported to be involved with inflammasome activation through ROS production, or through interaction with the components of the NLRP3 inflammasome. The role of mitochondrial ROS (mtROS) in NLRP3 inflammasome activation was originally proposed from a study showing that mtROS, as generated by the inhibition of the mitochondrial respiratory chain, can activate the NLRP3 inflammasome [97]. Similarly, Nakahira et al. found that mtROS generated from dysfunctional mitochondria is required for NLRP3 inflammasome activation in response to LPS and ATP, and mitochondrial DNA (mtDNA) is released into the cytosol in both an NLRP3- and mtROS-dependent manner [98]. Shimada et al. later found that, while mtDNA interacts with both NLRP3 and AIM2, oxidized mtDNA is specifically required for NLRP3 inflammasome activation [99]. Recently, Zhong et al. reported that newly synthesized mtDNA that is induced by TLR signaling is oxidized and required for NLRP3 inflammasome activation [47]. Taken together, these findings suggest a role for mitochondrial dysfunction, mtROS, and mtDNA in NLRP3 inflammasome activation. However, other studies questioned the role of mtROS and mitochondrial dysfunction in NLRP3 inflammasome activation [36,67,100]. Notably, most of these studies inhibit mtROS production and mitochondrial function with high concentrations of chemical inhibitors, which are prone to cause artifacts. At least one study reported that ROS inhibitors also block the priming signal for NLRP3 inflammasome activation [100]. Moreover, Munoz-Planillo et al. showed that mitochondrial dysfunction and mtROS production are dispensable in NLRP3 inflammasome activation [67].

Besides the generation of mtROS and mtDNA, mitochondria are shown to co-localize with the NLRP3 inflammasome. Mitochondrial molecules, which include mitochondrial antiviral-signaling protein (MAVS), mitofusin 2, and cardiolipin, associate with NLRP3 in response to NLRP3 stimuli. MAVS physically interacts with NLRP3 and it is required for NLRP3 inflammasome activation induced by soluble stimuli, such as ATP, nigericin, and poly (I:C), but not by particulate stimuli, such as silica, alum, and MSU [101,102]. However, other studies reported that MAVS is required for NLRP3 inflammasome activation by viral infections, but not by non-viral NLRP3 stimuli [101,103,104]. MAVS recruits TRAF3 to ASC in the context of viral infection, which ubiquitinates ASC promoting its oligomerization, and it therefore enhances NLRP3 inflammasome activation [105]. NLRP3 was also shown to directly associate with mitochondrial mitofusin 2 during viral infection [106]. Cardiolipin, which is a lipid of the inner mitochondrial membrane, was shown to externalize and bind directly to the LRR domain of NLRP3, and disrupting cardiolipin expression is detrimental to NLRP3 activation [107]. More recently, NLRP3 and caspase-1 were both found to associate with cardiolipin at the outer mitochondrial membrane following priming by TLR1/2, TLR3, and TLR4 agonists [108]. It was also suggested that NLRP3 stimuli induce the transport of mitochondria to the ER, where the ASC present on the mitochondria come into close proximity to NLRP3 on the ER [109]. These findings suggest that mitochondria may function as scaffolds for the assembly of the NLRP3 inflammasome. However, at least one study reported that both NLRP3 and caspase-1 co-localizes with the ASC pyroptosome in the cytosol, instead of on mitochondria or other major organelles [110]. Furthermore, a recent study found that the NLRP3 stimuli-induced dispersed trans-Golgi network serves as a scaffold for NLRP3 inflammasome activation, while, in contrast, mitochondria-associated NLRP3 fails to activate the inflammasome [111]. Another study also supports Golgi involvement in NLRP3 activation via protein kinase D signaling at the mitochondria-associated endoplasmic reticulum membranes [112]. Thus, future studies are required for clarifying the role of ROS and mitochondria in NLRP3 inflammasome activation.

### 3.3. Lysosomal Damage

Particulate matter, such as MSU, alum, silica, asbestos, amyloid-β, cholesterol crystals, and calcium crystals, induces NLRP3 inflammasome activation in macrophages [51,52,53,113,114,115,116]. Particulate matter damages lysosomes after phagocytosis, which results in the leakage of lysosomal contents into the cytosol. Lysosomal disruption appears to be a critical step for NLRP3 inflammasome activation by particulate matter, since rupturing lysosomes directly by Leu-Leu-OMe can trigger NLRP3 inflammasome activation [52]. However, the mechanism linking lysosomal disruption to NLRP3 inflammasome activation remains unclear. The H^+^ ATPase inhibitor bafilomycin A inhibits particulate matter-induced NLRP3 inflammasome activation, which suggests that lysosomal acidification is required in this process [52]. Similarly, it was proposed that inflammasome activation by MSU is reliant on the acidic conditions within lysosomes to cause massive Na^+^ release to increase cellular osmolarity and water influx, which results in decreased intracellular K^+^ concentration [86].

Additionally, it was hypothesized that active lysosomal enzymes are released into the cytosol to trigger NLRP3 inflammasome activation after the phagocytosis of particulate matter. Cathepsin B release from lysosomes was initially thought to be the candidate, as NLRP3 inflammasome activation by particulate matter is inhibited in macrophages that are treated with CA-074-Me, a chemical inhibitor for cathepsin B [52,115]. Furthermore, it was reported that lysosomal cathepsin B release is required for IL-1β release, but not for pro-IL-1β production, which suggests the involvement of cathepsin B in NLRP3 inflammasome activation [85]. Additionally, the protein α-synuclein in its fibrillar form, which known to contribute to Parkinson’s disease, induces NLRP3 inflammasome activation by disrupting the lysosome and facilitating the release of cathepsin B [117]. However, macrophages from the cathepsin B-deficient mice show comparable NLRP3 inflammasome activation as wild-type cells in response to particulate matter, suggesting that the inhibition of NLRP3 inflammasome activation by CA-074-Me might be an off-target effect [118]. Alternatively, the other members of the cathepsin family might contribute to NLRP3 inflammasome activation in cathepsin B-deficient macrophages. Indeed, cathepsins B, L, C, S, and X were revealed to have redundant roles in NLRP3 inflammasome activation by particulate matter, while cathepsin X alone plays a significant role in cell death that is induced by nigericin [119]. Additionally, a study has shown that the release of lysosomal contents is key to inflammasome activation in group B *Streptococcus* infection; however, whether cathepsin B or other factors, such as bacterial RNA, were the activating agents remains unclear [55]. In adenovirus infection, cathepsin B release is also required, although the inhibition of ROS attenuates IL-1β secretion, which suggests that cathepsin B may induce inflammasome activation through the promotion of ROS [120]. These studies support a role for cathepsin B for the activation of NLRP3, although further studies will be required to determine the mechanism by which lysosomal damage activates the NLRP3 inflammasome.

## 4. Activation of the Non-Canonical Inflammasome Pathway and Alternative Inflammasome Pathway

### 4.1. The Non-Canonical Inflammasome Pathway

**Figure 2:**
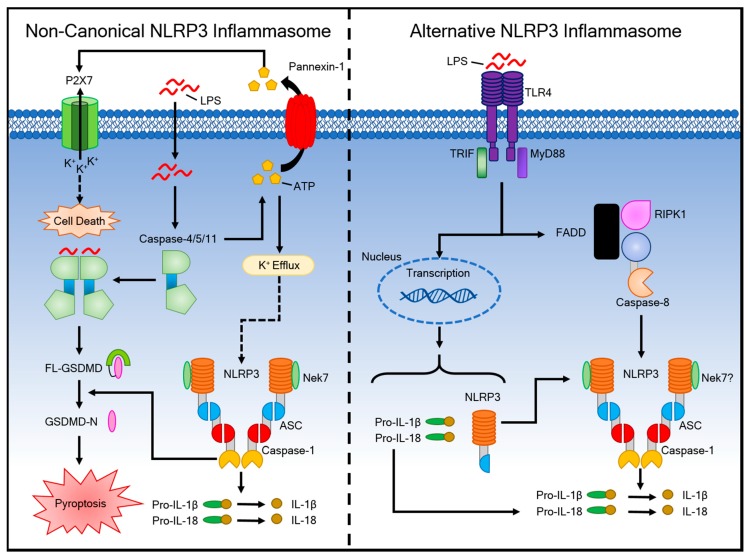
Mechanism of Activation for the Non-Canonical and Alternative NLRP3 Inflammasome Pathways. Non-canonical NLRP3 inflammasome activation (**left**) is induced by LPS internalization into the cytosol by transfection or infection. Caspase-11/4/5 induces pyroptosis through the cleavage of GSDMD. This process also activates pannexin-1 through caspase-11 to release ATP and induce K^+^ efflux, which drives NLRP3 inflammasome assembly and release of IL-1β. The alternative NLRP3 inflammasome (**right**) is activated in human monocytes in response to LPS and requires receptor-interacting serine/threonine-protein kinase 1 (RIPK1), FADD, and caspase-8 for its activation. This pathway is K^+^ efflux independent and does not induce pyroptosis.

It was discovered that cytoplasmic LPS is sufficient for eliciting endotoxic shock independently of TLR4 signaling [121,122]. This pathway is called the non-canonical inflammasome and it responds to Gram-negative but not Gram-positive bacteria (Figure 2).

This non-canonical inflammasome represents an additional layer of defense, which is pertinent to pathogens that have evolved to bypass cell surface TLR4 [123]. The non-canonical inflammasome involves caspases 4/5 in humans and caspase-11 in mice, rather than caspase-1 [124,125]. These caspases sense intracellular LPS independently of TLR4 by directly binding to LPS [126]. The component of LPS that is sensed by these noncanonical caspases is pent-acylated and hexa-acylated lipid A, but not tetra-acylated lipid A, which is the reason why some bacteria, such as *Francisella*, can survive in the cytosol [121]. Priming, as with the canonical pathway, enhances the inflammatory response in mice due to the low basal expression of caspase-11 [121]. In contrast, priming is unnecessary for non-canonical inflammasome activation in human cells that express high levels of caspase-4 [126]. TLR4-dependent and TRIF-dependent IFN-α/β production are required for caspase-11 activation in macrophages, and they are partially required for pro-caspase-11 expression [127]. Interestingly, mice lacking caspase-1 gene expression (Casp1^−/−^), but not caspase-11 gene expression, are more susceptible to *Salmonella* infection than mice lacking both caspase-1 and caspase-11 [127]. Caspases-4/5/11 induce pyroptosis through the processing of GSDMD, and pannexin-1, which is a protein channel that releases ATP from the cell [17,71,128]. This extracellular ATP activates the P2X7 receptor (P2X7R) [129], an ATP-gated cation selective channel that opens a pore that triggers K^+^ efflux [130]. Oxidized phospholipid 1-palmitoyl-2-arachidonoyl-sn-glycero-3-phosphorylcholine (oxPAPC) inhibits LPS-induced pyroptosis through the non-canonical inflammasome by directly binding to caspase-4 or caspase-11 in macrophages, where it competes with LPS binding [131]. A recent study showed that, in mice, GBP2 and IRGB10 liberate bacterial ligands to promote caspase-11 activation in pathogenic *P. aeruginosa* populations that lack a type 3 secretion system [123]. These proteins were reported to act together to cause bacterial cell membrane instability, resulting in the release of these bacterial ligands for recognition by caspase-11 [132,133].

### 4.2. The Alternative Inflammasome Pathway

An alternative pathway was observed to function unlike either the canonical or non-canonical pathways (Figure 2). Human monocytes do not require secondary stimuli following LPS stimulation to activate caspase-1 and induce IL-1β maturation and secretion [129,134]. The alternative inflammasome pathway does not require K^+^ efflux, induce ASC speck formation, or lead to subsequent pyroptosis [135]. In murine dendritic cells, prolonged exposure to LPS, without other activating signals, resulted in NLRP3-mediated IL-1β processing and secretion in a P2X7 independent manner [136]. TLR4-TRIF-RIPK1-FADD-CASP8 signaling is involved in this alternative pathway [135].

## 5. Regulation of the NLRP3 Inflammasome

**Figure 3:**
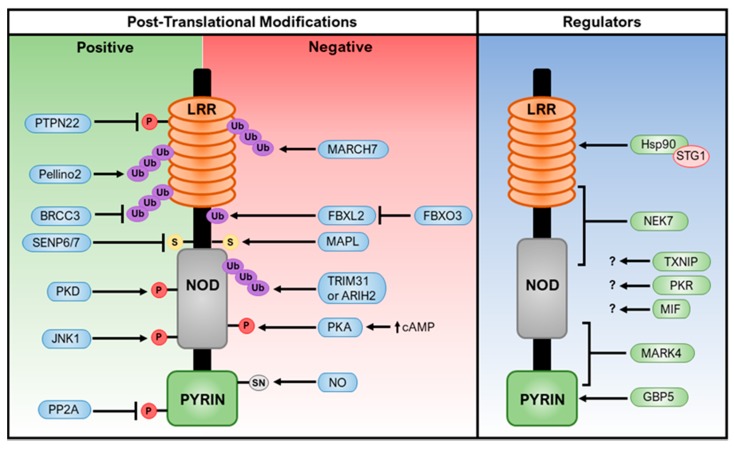
Post-translational Modifications and Regulators of NLRP3. NLRP3 is regulated via phosphorylation (P), ubiquitination (Ub), sumolyation (S), and s-nitrosylation (SN) through post-translational modifications. Post-translational modifications that positively affect NLRP3 activation are listed on the left and those that negatively act on NLRP3 to inhibit its activation are listed in the middle. Interacting partners of NLRP3 are listed on the right. ARIH2, ariadne homolog 2; BRCC3, BRCA1/BRCA2-containing complex subunit 3; FBX12, F-box/LRR-repeat protein 2; FBXO3, F-box only protein 3; JNK1, JUN N-terminal kinase 1; MAPL, mitochondrial-anchored protein ligase; MARCH7, membrane-associated RING finger protein 7; NO, nitric oxide; PKA, protein kinase A; PKD, protein kinase D; PP2A, phosphatase 2A; PTPN22, protein tyrosine phosphatase non-receptor 22; SENP6/7, sentrin/SUMO-specific proteases. GBP5, guanylate-binding protein 5; Hsp90, heat-shock protein 90; MARK4, microtubule-affinity regulating kinase 4; MIF, macrophage migration inhibitory factor; NEK7, NIMA-related kinase 7; PKR, double-stranded RNA-dependent protein kinase; STG1, suppressor of the G2 allele of skp1; TXNIP, thioredoxin-interacting protein.

The activation of the NLRP3 inflammasome contributes to host defense against microbial infections. However, when dysregulated, the NLRP3 inflammasome is implicated in the pathogenesis of several inflammatory disorders. Therefore, it is critical that NLRP3 inflammasome activation is precisely regulated to provide adequate immune protection without causing damage to the host tissues. Indeed, several mechanisms, including post-translational modifications of NLRP3 and interacting partners, have been identified to regulate NLRP3 inflammasome activation (Figure 3).

### 5.1. Regulation by Post-Translational Modifications of NLRP3

Multiple post-translational modifications of NLRP3 have been identified in regulating NLRP3 inflammasome activation [137]. Of these modifications, the ubiquitination and phosphorylation of NLRP3 are the best characterized.

#### 5.1.1. Ubiquitination

Lopez-Castejon et al. first reported that NLRP3 is ubiquitinated and inhibition of its deubiquitination by a DUB inhibitor b-AP15 blocks NLRP3 inflammasome activation [44]. Similarly, Py et al. found that a DUB/isopeptidase inhibitor G5 also specifically suppresses the activation of the NLRP3 inflammasome, and further identified protein BRCC3 (mouse)/BRCC36 (human) as the enzyme that is responsible for the deubiquitinating of NLRP3 during priming [45]. BRCC3 specifically cleaves K63-linked, but not K48-linked, polyubiquitin chains. These studies suggested an inhibitory role for NLRP3 ubiquitination in NLRP3 inflammasome activation. A later study identified the Skp-Cullin-F box (SCF) family member, F-box L2 (FBXL2), as an E3 ubiquitin ligase for NLRP3 [138]. FBXL2 mediates NLRP3 ubiquitination and proteasomal degradation. Interestingly, itself, FBXL2 is also regulated by another F-box protein, F-box O3 (FBXO3), through ubiquitination and degradation [138]. Additionally, a ubiquitin ligase MARCH7, which is activated downstream of the dopamine D1 receptor DRD1 pathway, mediates the K48-linked polyubiquitination of NLRP3 and inhibits NLRP3 inflammasome activation [139]. Similarly, TRIM31, another priming-induced E3 ubiquitin ligase, was shown to directly interact with NLRP3 and promote its K48-linked ubiquitination and subsequent proteasomal degradation [140]. Moreover, ariadne homolog 2 (ARIH2) ubiquitinates the NACHT domain of NLRP3 and inhibits NLRP3 inflammasome activation [141]. A recent study reported that Pellino2 promotes NLRP3 inflammasome activation by inducing the K63-linked ubiquitination of NLRP3 during the priming phase, in contrast to the inhibitory role of NLRP3 ubiquitination [142]. Deubiquitinases USP7 and USP47 positively regulate NLRP3 inflammasome formation through their redundant function in promoting ASC oligomerization and speck formation [143]. USP7 and USP47 also regulate NLRP3 ubiquitination status, although the exact mechanism is yet to be elucidated [143]. Pellino2, USP7, and USP47 only regulate canonical inflammasome activation [142,143]. Therefore, NLRP3 ubiquitination has either a negative or positive role in NLRP3 inflammasome activation, depending on the ubiquitin ligase and type of ubiquitination.

#### 5.1.2. Phosphorylation

Protein phosphorylation is a common mechanism that is involved in many signaling pathways, including NLRP3 inflammasome activation [144]. Mortimer et al. reported that protein kinase A (PKA) phosphorylates human NLRP3 at the Ser295 (Ser291 for mouse NLRP3) and this phosphorylation negatively regulates NLRP3 inflammasome activation through the inhibition of NLRP3 ATPase activity [145]. Guo et al. showed a different inhibitory mechanism of NLRP3 phosphorylation at the same site [146]. In this case, bile acids that are derived from cholesterol metabolism activate the TGR5 receptor pathway, which results in the increase of intracellular cAMP and the subsequent activation of PKA. Activated PKA was shown to interact with NLRP3 and phosphorylate Ser291 (mouse NLRP3) in the NOD domain of NLRP3. NLRP3 phosphorylation promotes its K48- and K63- linked polyubiquitination and subsequent degradation [146]. Interestingly, Zhang et al. reported that NLRP3 phosphorylation at the same residue promoted NLRP3 inflammasome activation [112]. In this study, NLRP3 stimuli induced the translocation of mitochondria-associated membrane (MAM) to the adjacent Golgi membrane, where the accumulation of diacylglycerol (DAG) occurs. DAG activates protein kinase D, which subsequently phosphorylates human NLRP3 at Ser295. This phosphorylation of NLRP3 promotes the assembly of the NLRP3 inflammasome complex [112]. Currently, it is unclear why NLRP3 phosphorylation at the same site has opposing functions in NLRP3 inflammasome activation. Priming signals also induces NLRP3 phosphorylation. Song et al. reported that priming signals induce JNK1-mediated NLRP3 phosphorylation at Ser194, which is required for NLRP3 deubiquitination and activation [46]. NLRP3 phosphorylation was also implicated in playing an inhibitory role for NLRP3 activation, which suggests a potential role for protein phosphatases in the regulation of NLRP3 inflammasome activation. Indeed, PTPN22 was shown to interact with and dephosphorylate NLRP3 at Tyr861, which is required for NLRP3 inflammasome activation [147]. In addition, PP2A was found to dephosphorylate Ser5 (human NLRP3) of NLRP3 and promote NLRP3 inflammasome activation [148].

#### 5.1.3. Other Post-Translational Modifications

Besides ubiquitination and phosphorylation, other PTMs of NLRP3 were reported to regulate NLRP3 inflammasome activation. Several studies reported that the nitrosylation of NLRP3 blocks its activity [149,150,151]. For example, NLRP3 was shown to be S-nitrosylated in *Mycobacterium tuberculosis*-infected macrophages, and this s-nitrosylation inhibits NLRP3 inflammasome assembly [151]. This s-nitrosylation is induced by IFN-γ, which provides a mechanism for NLRP3 inflammasome regulation by adaptive immunity [151]. Moreover, sumoylation negatively regulates the NLRP3 inflammasome. SUMO E3-ligase MAPL (a.k.a. Mul1) targets NLRP3, and that, upon stimulation, SUMO-specific proteases SENP6 and SENP7 desumoylate NLRP3, which promotes NLRP3 inflammasome activation [152]. In addition, ADP-ribosylation of NLRP3 by *Mycoplasma pneumonia* promotes NLRP3 inflammasome activation [153].

### 5.2. Regulation by NLRP3 Interacting Partners

Multiple NLRP3-interacting proteins, which include the molecular chaperone heat shock protein 90 (Hsp90) and its co-chaperone SGT1, thioredoxin-interacting protein (TXNIP), guanylate-binding protein 5 (GBP5), double-stranded RNA-dependent protein kinase (PKR), migration inhibitory factor (MIF), microtubule-affinity regulating kinase 4 (MARK4), and Nek7, have been reported to regulate the NLRP3 inflammasome. Hsp90 is required to protect NLRP3 from degradation by both the proteasome and autophagy [154,155]. Hsp90 recruits SGT1 to NLRP3 to form a complex, which was proposed to maintain NLRP3 in an inactive, but signaling-competent, state [154]. The pharmacological inhibition of Hsp90 or siRNA-mediated SGT1 depletion attenuates NLRP3 inflammasome activation [154,155,156,157]. Of note, Hsp90 and SGT1 also interact with other NLRs and regulate their activity, including NOD2 and NLRC4 [154]. TXNIP, as an oxidative sensor, interacts with thioredoxin (TRX) under reducing conditions. ROS induced by NLRP3 stimuli oxidizes TRX and it causes the dissociation of TXNIP from TRX, leading to the interaction of TXNIP with NLRP3 and subsequent NLRP3 inflammasome activation [158]. However, a different study found that TXNIP is dispensable for NLRP3 inflammasome activation that is induced by ATP, MSU, and islet amyloid polypeptide [159]. Although the exact reason for those different conclusions is unknown, NLRP3 stimuli and cell types used in those studies might contribute to the discrepancy. As one of the genes that is inducible by LPS or IFN-γ, guanylate binding protein 5 (GBP5) was reported to be critical for NLRP3 inflammasome activation in response to ATP, nigericin, and pathogenic bacteria, but not particulate matter [160]. GBP5 binds to the pyrin domain of NLRP3 and tetrameric GBP5 promotes NLRP3-mediated ASC oligomerization [160]. However, other studies reported that GBP5 is dispensable for the NLRP3 inflammasome activation in macrophages [133,161]. Thus far, most of the studies support the role of GBP5 in the activation of the AIM2 inflammasome, but not the NLRP3 inflammasome, and further studies are required for clarifying the role of GBP5 in NLRP3 inflammasome activation [161,162]. Lu et al. reported that PKR regulates the activation of several inflammasomes, including the NLRP1, NLRP3, NLRC4, and AIM2 inflammasomes [163]. The deletion or inhibition of PKR reduces inflammasome-mediated activation of caspase-1 and maturation of IL-1β and IL-18. Yoshida et al. recently reported that PKR indirectly regulates inflammasome activation in osteoblasts by regulating the NF-κB pathway [164]. However, one study showed that the activation of inflammasomes is not affected in the macrophages from two different PKR-deficient mice, including the one that was used in the initial study [165]. Additionally, other NLRP3-interacting proteins, including MIF and MARK4, were reported to promote NLRP3 inflammasome activation [166,167]. Further studies are required for confirming the roles of those NLRP3-interacting protein in NLRP3 inflammasome activation.

Recently, three groups independently showed that Nek7 is a critical regulator for NLRP3 inflammasome activation [168,169,170]. Nek7, as the smallest member of the NIMA-related kinases family, plays a role in the mitotic progression and DNA damage response and it is critical for embryo development and survival [171]. Nek7 is required for the activation of the NLRP3 inflammasome, but it is dispensable for the activation of NLRC4 or AIM2 inflammasome. The catalytic domain of Nek7, but not its kinase activity, is required for its interaction with the NOD and LRR domains of NLRP3 and inflammasome activation [168,170]. Mechanistically, Nek7 promotes NLRP3 oligomerization, ASC speck formation, and caspase-1 activation downstream of potassium efflux [168]. Furthermore, Nek7 is also required for NLRP3 inflammasome activation in macrophages that harbor an NLRP3 activating mutation (NLRP3^R258W^) and by K^+^-efflux independent stimuli, such as imiquimod and CL097 [72,168]. Three *in vivo* models have further demonstrated the critical role for Nek7 in NLRP3 inflammasome activation [168,170]. Therefore, these studies clearly show that Nek7 is critical in NLRP3 inflammasome activation; however, the mechanism by which Nek7 regulates NLRP3 inflammasome activation remains to be determined. Since all of the tested NLRP3 stimuli, whether they induce K^+^ efflux or not, and NLRP3 activating mutation require Nek7 to activate the NLRP3 inflammasome, Nek7 might act as proximal regulator for NLRP3 oligomerization and the assembly of the NLRP3 inflammasome. Understanding the mechanism by which Nek7 regulates NLRP3 activation will provide new insight into the molecular mechanism of NLRP3 inflammasome activation and regulation.

## 6. Concluding Remarks and Perspectives

The NLRP3 inflammasome has been the most intensively investigated inflammasome in the past decade. However, a unified mechanism for NLRP3 inflammasome activation has not yet emerged from the field. NLRP3 stimuli induce multiple signaling and cellular events and are shown to trigger NLRP3 inflammasome activation. Among these events, K^+^ efflux remains the critical signaling event that is required for NLRP3 inflammasome activation by most stimuli, while the role of other events, such as Ca^2+^ mobilization, Cl^−^ efflux, ROS, and mitochondrial dysfunction remains to be clarified. Recently, the identification of Nek7 as a critical NLRP3 regulator, as well as new findings on the roles of a dispersed trans-Golgi network and newly synthesized mtDNA in NLRP3 inflammasome activation, represents major advances in the field. However, future studies are needed to understand the mechanism by which Nek7 regulates NLRP3 inflammasome activation and how NLRP3 stimuli-induced signaling events are integrated to activate the NLRP3 inflammasome.

## Abbreviations

PRR	Pattern-recognition receptor
PAMP	Pathogen-associated molecular pattern
DAMP	Damage-associated molecular pattern
PYD	Pyrin domain
NOD	Nucleotide-binding oligomerization domain
LRR	Leucine-rich repeat
NLR	Nod-like receptor
NLRP	Nod-like receptor protein
NLRP3	NLR family pyrin domain containing 3
NLRC4	NLR family CARD domain-containing protein 4
AIM2	Absent-in-melanoma 2
ASC	Apoptosis-associated speck-like protein containing a caspase-recruitment domain
IL	Interleukin
IFN	Interferon
GSDMD	Gasdermin D
CAPS	Cryopyrin-associated periodic syndromes
IL-1R	IL-1 receptor
MCC950	1-(1,2,3,5,6,7-Hexahydro-s-indacen-4-yl)-3-[4-(2-hydroxypropan-2-yl)furan-2-yl]sulfonylurea
PtdIns4	Phosphatidylinositol-4-phosphate
ATP	Adenosine triphosphate
TLR	Toll-like receptors
NF-κB	Nuclear factor kappa-light-chain-enhancer of activated B cells
MYD88	Myeloid differentiation primary response 88
TRIF	TIR-domain-containing adapter-inducing interferon-β
FADD	Fas-associated protein with death domain
LPS	Lipopolysaccharide
IRAK	IL-1 receptor-associated kinase
IKK	Inhibitor of NF-κB kinase
BRCC	BRCA1-BRCA2-containing complex
JNK	c-Jun N-terminal kinase
IRF1	Interferon regulatory transcription factor 1
mtDNA	Mitochondrial DNA
PLC	Phospholipase C
CL097	2-(ethoxymethyl)-3H-imidazo[4,5-c]quinolin-4-amine
BAPTA-AM	1,2-Bis(2-aminophenoxy)ethane-N,N,N′,N′-tetraacetic acid tetrakis(acetoxymethyl ester)
NADPH	Nicotinamide adenine dinucleotide phosphate
GPCR	G protein-coupled receptor
CaSR	Calcium-sensing receptor
GPRC6A	G protein-coupled receptor family C group 6 member A
PIP2	Phosphatidylinositol 4,5-bisphosphate
IP3	Inositol 1,4,5-triphosphate
ER	Endoplasmic reticulum
IP3R	IP3 receptor
2APB	2-aminoethoxy diphenylborinate
P2RX7	P2X purinoceptor 7
TRPM	Transient receptor potential ion melastatin
TRPV	Transient receptor potential ion vanilloid
TXNIP	Thioredoxin interacting protein
MSU	Monosodium urate
VRAC	Volume-related anion channel
CLIC	Chloride intracellular channel
ROS	Reactive oxygen species
NOX2	NADPH oxidase 2
NOX4	NADPH oxidase 4
CPT1A	Carnitine palmitoyltransferase 1A
mtROS	Mitochondrial ROS
MAVS	Mitochondrial antiviral-signaling protein
TRAF3	TNF receptor associated factor 3
oxPAPC	Oxidized phospholipid 1-palmitoyl-2-arachidonoyl-sn-glycero-3-phosphorylcholine
GBP2	Guanylate-binding protein 2
IRGB10	Immunity-related GTPase family member b10
RIPK1	Receptor-interacting serine/threonine-protein kinase 1
CASP8	Caspase-8
DUB	Deubiquitinating enzyme
SCF	Skp-Cullin-F box
FBXL2	F-box L2
FBXO3	F-box O3
MARCH7	Membrane-associated RING-CH protein VII
DRD1	Dopamine D1 receptor
TRIM31	Tripartite Motif Containing 31
ARIH2	Ariadne homolog 2
USP	Ubiquitin specific peptidase
PKA	Protein kinase A
cAMP	Cyclic AMP
MAM	Mitochondria-associated membrane
DAG	Diacylglycerol
PTPN22	Protein tyrosine phosphatase, non-receptor type 22
PP2A	Phosphotase 2A
MAPL	Mitochondrial-anchored protein ligase
SENP	Sentrin/SUMO-specific protease
HSP90	Heat shock protein 90
SGT1	Suppressor of G2 allele of SKP1
TXNIP	Thioredoxin-interacting protein
GBP5	Guanylate-binding protein 5
PKR	Double-stranded RNA-dependent protein kinase
MIF	Migration inhibitory factor
MARK4	Microtubule-affinity regulating kinase 4
Nek7	NIMA-related kinase 7

## References

[1] Takeuchi O., Akira S. (2010). Pattern recognition receptors and inflammation. Cell.

[2] Franchi L., Eigenbrod T., Muñoz-Planillo R., Nuñez G. (2009). The Inflammasome: A Caspase-1 Activation Platform Regulating Immune Responses and Disease Pathogenesis. Nat. Immunol..

[3] Sharma D., Kanneganti T.-D. (2016). The cell biology of inflammasomes: Mechanisms of inflammasome activation and regulation. J. Cell Biol..

[4] Lamkanfi M., Dixit V.M. (2014). Mechanisms and Functions of Inflammasomes. Cell.

[5] Elinav E., Strowig T., Kau A.L., Henao-Mejia J., Thaiss C.A., Booth C.J., Peaper D.R., Bertin J., Eisenbarth S.C., Gordon J.I. (2011). NLRP6 inflammasome regulates colonic microbial ecology and risk for colitis. Cell.

[6] Kerur N., Veettil M.V., Sharma-Walia N., Bottero V., Sadagopan S., Otageri P., Chandran B. (2011). IFI16 acts as a nuclear pathogen sensor to induce the inflammasome in response to Kaposi Sarcoma-associated herpesvirus infection. Cell Host Microbe.

[7] Khare S., Dorfleutner A., Bryan N.B., Yun C., Radian A.D., de Almeida L., Rojanasakul Y., Stehlik C. (2012). An NLRP7-containing inflammasome mediates recognition of microbial lipopeptides in human macrophages. Immunity.

[8] Minkiewicz J., de Rivero Vaccari J.P., Keane R.W. (2013). Human astrocytes express a novel NLRP2 inflammasome. Glia.

[9] Vladimer G.I., Weng D., Paquette S.W.M., Vanaja S.K., Rathinam V.A.K., Aune M.H., Conlon J.E., Burbage J.J., Proulx M.K., Liu Q. (2012). The NLRP12 inflammasome recognizes Yersinia pestis. Immunity.

[10] Fernandes-Alnemri T., Wu J., Yu J.-W., Datta P., Miller B., Jankowski W., Rosenberg S., Zhang J., Alnemri E.S. (2007). The pyroptosome: A supramolecular assembly of ASC dimers mediating inflammatory cell death via caspase-1 activation. Cell Death Differ..

[11] Manji G.A., Wang L., Geddes B.J., Brown M., Merriam S., Al-Garawi A., Mak S., Lora J.M., Briskin M., Jurman M. (2002). PYPAF1, a PYRIN-containing Apaf1-like protein that assembles with ASC and regulates activation of NF-kappa B. J. Biol. Chem..

[12] Franchi L., Warner N., Viani K., Nuñez G. (2009). Function of Nod-like receptors in microbial recognition and host defense. Immunol. Rev..

[13] Martinon F., Burns K., Tschopp J. (2002). The Inflammasome: A Molecular Platform Triggering Activation of Inflammatory Caspases and Processing of proIL-β. Mol. Cell.

[14] Dinarello C.A. (2009). Immunological and Inflammatory Functions of the Interleukin-1 Family. Annu. Rev. Immunol..

[15] Fink S.L., Cookson B.T. (2006). Caspase-1-dependent pore formation during pyroptosis leads to osmotic lysis of infected host macrophages. Cell. Microbiol..

[16] Shi J., Zhao Y., Wang K., Shi X., Wang Y., Huang H., Zhuang Y., Cai T., Wang F., Shao F. (2015). Cleavage of GSDMD by inflammatory caspases determines pyroptotic cell death. Nature.

[17] Kayagaki N., Stowe I.B., Lee B.L., O’Rourke K., Anderson K., Warming S., Cuellar T., Haley B., Roose-Girma M., Phung Q.T. (2015). Caspase-11 cleaves gasdermin D for non-canonical inflammasome signalling. Nature.

[18] He W., Wan H., Hu L., Chen P., Wang X., Huang Z., Yang Z.-H., Zhong C.-Q., Han J. (2015). Gasdermin D is an executor of pyroptosis and required for interleukin-1β secretion. Cell Res..

[19] Miao E.A., Leaf I.A., Treuting P.M., Mao D.P., Dors M., Sarkar A., Warren S.E., Wewers M.D., Aderem A. (2010). Caspase-1-induced pyroptosis is an innate immune effector mechanism against intracellular bacteria. Nat. Immunol..

[20] Thomas P.G., Dash P., Aldridge J.R., Ellebedy A.H., Reynolds C., Funk A.J., Martin W.J., Lamkanfi M., Webby R.J., Boyd K.L. (2009). The intracellular sensor NLRP3 mediates key innate and healing responses to influenza A virus via the regulation of caspase-1. Immunity.

[21] Allen I.C., Scull M.A., Moore C.B., Holl E.K., McElvania-TeKippe E., Taxman D.J., Guthrie E.H., Pickles R.J., Ting J.P.-Y. (2009). The NLRP3 inflammasome mediates in vivo innate immunity to influenza A virus through recognition of viral RNA. Immunity.

[22] Gross O., Poeck H., Bscheider M., Dostert C., Hannesschläger N., Endres S., Hartmann G., Tardivel A., Schweighoffer E., Tybulewicz V. (2009). Syk kinase signalling couples to the Nlrp3 inflammasome for anti-fungal host defence. Nature.

[23] Kanneganti T.-D., Body-Malapel M., Amer A., Park J.-H., Whitfield J., Franchi L., Taraporewala Z.F., Miller D., Patton J.T., Inohara N. (2006). Critical role for Cryopyrin/Nalp3 in activation of caspase-1 in response to viral infection and double-stranded RNA. J. Biol. Chem..

[24] Menu P., Vince J.E. (2011). The NLRP3 inflammasome in health and disease: The good, the bad and the ugly. Clin. Exp. Immunol..

[25] Guo H., Callaway J.B., Ting J.P.-Y. (2015). Inflammasomes: Mechanism of action, role in disease, and therapeutics. Nat. Med..

[26] Vajjhala P.R., Mirams R.E., Hill J.M. (2012). Multiple binding sites on the pyrin domain of ASC protein allow self-association and interaction with NLRP3 protein. J. Biol. Chem..

[27] Duncan J.A., Bergstralh D.T., Wang Y., Willingham S.B., Ye Z., Zimmermann A.G., Ting J.P.-Y. (2007). Cryopyrin/NALP3 binds ATP/dATP, is an ATPase, and requires ATP binding to mediate inflammatory signaling. Proc. Natl. Acad. Sci. USA.

[28] Coll R.C., Hill J.R., Day C.J., Zamoshnikova A., Boucher D., Massey N.L., Chitty J.L., Fraser J.A., Jennings M.P., Robertson A.A.B. (2019). MCC950 directly targets the NLRP3 ATP-hydrolysis motif for inflammasome inhibition. Nat. Chem. Biol..

[29] Coll R.C., Robertson A.A.B., Chae J.J., Higgins S.C., Muñoz-Planillo R., Inserra M.C., Vetter I., Dungan L.S., Monks B.G., Stutz A. (2015). A small-molecule inhibitor of the NLRP3 inflammasome for the treatment of inflammatory diseases. Nat. Med..

[30] Tapia-Abellán A., Angosto-Bazarra D., Martínez-Banaclocha H., Torre-Minguela C.d., Cerón-Carrasco J.P., Pérez-Sánchez H., Arostegui J.I., Pelegrin P. (2019). MCC950 closes the active conformation of NLRP3 to an inactive state. Nat. Chem. Biol..

[31] Hafner-Bratkovič I., Sušjan P., Lainšček D., Tapia-Abellán A., Cerović K., Kadunc L., Angosto-Bazarra D., Pelegrίn P., Jerala R. (2018). NLRP3 lacking the leucine-rich repeat domain can be fully activated via the canonical inflammasome pathway. Nat. Commun..

[32] Lamkanfi M., Kanneganti T.-D. (2010). Nlrp3: An immune sensor of cellular stress and infection. Int. J. Biochem. Cell Biol..

[33] Bauernfeind F.G., Horvath G., Stutz A., Alnemri E.S., MacDonald K., Speert D., Fernandes-Alnemri T., Wu J., Monks B.G., Fitzgerald K.A. (2009). Cutting Edge: NF-κB Activating Pattern Recognition and Cytokine Receptors License NLRP3 Inflammasome Activation by Regulating NLRP3 Expression. J. Immunol..

[34] Franchi L., Eigenbrod T., Núñez G. (2009). Cutting Edge: TNF-α Mediates Sensitization to ATP and Silica via the NLRP3 Inflammasome in the Absence of Microbial Stimulation. J. Immunol..

[35] Gurung P., Anand P.K., Malireddi R.K.S., Walle L.V., Opdenbosch N.V., Dillon C.P., Weinlich R., Green D.R., Lamkanfi M., Kanneganti T.-D. (2014). FADD and Caspase-8 Mediate Priming and Activation of the Canonical and Noncanonical Nlrp3 Inflammasomes. J. Immunol..

[36] Allam R., Lawlor K.E., Yu E.C.-W., Mildenhall A.L., Moujalled D.M., Lewis R.S., Ke F., Mason K.D., White M.J., Stacey K.J. (2014). Mitochondrial apoptosis is dispensable for NLRP3 inflammasome activation but non-apoptotic caspase-8 is required for inflammasome priming. EMBO Rep..

[37] Lemmers B., Salmena L., Bidère N., Su H., Matysiak-Zablocki E., Murakami K., Ohashi P.S., Jurisicova A., Lenardo M., Hakem R. (2007). Essential Role for Caspase-8 in Toll-like Receptors and NFκB Signaling. J. Biol. Chem..

[38] Ranjan K., Pathak C. (2016). FADD regulates NF-κB activation and promotes ubiquitination of cFLIP_L_ to induce apoptosis. Sci. Rep..

[39] Juliana C., Fernandes-Alnemri T., Kang S., Farias A., Qin F., Alnemri E.S. (2012). Non-transcriptional Priming and Deubiquitination Regulate NLRP3 Inflammasome Activation. J. Biol. Chem..

[40] Schroder K., Sagulenko V., Zamoshnikova A., Richards A.A., Cridland J.A., Irvine K.M., Stacey K.J., Sweet M.J. (2012). Acute lipopolysaccharide priming boosts inflammasome activation independently of inflammasome sensor induction. Immunobiology.

[41] Lin K.-M., Hu W., Troutman T.D., Jennings M., Brewer T., Li X., Nanda S., Cohen P., Thomas J.A., Pasare C. (2014). IRAK-1 bypasses priming and directly links TLRs to rapid NLRP3 inflammasome activation. Proc. Natl. Acad. Sci. USA.

[42] Fernandes-Alnemri T., Kang S., Anderson C., Sagara J., Fitzgerald K.A., Alnemri E.S. (2013). Toll-Like Receptor Signaling Licenses IRAK1 For Rapid Activation Of The NLRP3 Inflammasome. J. Immunol. Baltim. Md 1950.

[43] Kim S.-J., Cha J.-Y., Kang H.S., Lee J.-H., Lee J.Y., Park J.-H., Bae J.-H., Song D.-K., Im S.-S. (2016). Corosolic acid ameliorates acute inflammation through inhibition of IRAK-1 phosphorylation in macrophages. BMB Rep..

[44] Lopez-Castejon G., Luheshi N.M., Compan V., High S., Whitehead R.C., Flitsch S., Kirov A., Prudovsky I., Swanton E., Brough D. (2013). Deubiquitinases regulate the activity of caspase-1 and interleukin-1β secretion via assembly of the inflammasome. J. Biol. Chem..

[45] Py B.F., Kim M.-S., Vakifahmetoglu-Norberg H., Yuan J. (2013). Deubiquitination of NLRP3 by BRCC3 critically regulates inflammasome activity. Mol. Cell.

[46] Song N., Liu Z.-S., Xue W., Bai Z.-F., Wang Q.-Y., Dai J., Liu X., Huang Y.-J., Cai H., Zhan X.-Y. (2017). NLRP3 Phosphorylation Is an Essential Priming Event for Inflammasome Activation. Mol. Cell.

[47] Zhong Z., Liang S., Sanchez-Lopez E., He F., Shalapour S., Lin X., Wong J., Ding S., Seki E., Schnabl B. (2018). New mitochondrial DNA synthesis enables NLRP3 inflammasome activation. Nature.

[48] Mariathasan S., Weiss D.S., Newton K., McBride J., O’Rourke K., Roose-Girma M., Lee W.P., Weinrauch Y., Monack D.M., Dixit V.M. (2006). Cryopyrin activates the inflammasome in response to toxins and ATP. Nature.

[49] Silveira A.A., Cunningham C., Corr E., Ferreira W.A., Costa F.F., Almeida C.B., Conran N., Dunne A. (2016). Heme Induces NLRP3 Inflammasome Formation in Primary Human Macrophages and May Propagate Hemolytic Inflammatory Processes By Inducing S100A8 Expression. Blood.

[50] Erdei J., Tóth A., Balogh E., Nyakundi B.B., Bányai E., Ryffel B., Paragh G., Cordero M.D., Jeney V. (2018). Induction of NLRP3 Inflammasome Activation by Heme in Human Endothelial Cells. Oxid. Med. Cell. Longev..

[51] Martinon F., Pétrilli V., Mayor A., Tardivel A., Tschopp J. (2006). Gout-associated uric acid crystals activate the NALP3 inflammasome. Nature.

[52] Hornung V., Bauernfeind F., Halle A., Samstad E.O., Kono H., Rock K.L., Fitzgerald K.A., Latz E. (2008). Silica crystals and aluminum salts activate the NALP3 inflammasome through phagosomal destabilization. Nat. Immunol..

[53] Dostert C., Pétrilli V., Bruggen R.V., Steele C., Mossman B.T., Tschopp J. (2008). Innate Immune Activation Through Nalp3 Inflammasome Sensing of Asbestos and Silica. Science.

[54] Eigenbrod T., Dalpke A.H. (2015). Bacterial RNA: An Underestimated Stimulus for Innate Immune Responses. J. Immunol..

[55] Gupta R., Ghosh S., Monks B., DeOliveira R.B., Tzeng T.-C., Kalantari P., Nandy A., Bhattacharjee B., Chan J., Ferreira F. (2014). RNA and β-hemolysin of group B Streptococcus induce interleukin-1β (IL-1β) by activating NLRP3 inflammasomes in mouse macrophages. J. Biol. Chem..

[56] Kanneganti T.-D., Ozören N., Body-Malapel M., Amer A., Park J.-H., Franchi L., Whitfield J., Barchet W., Colonna M., Vandenabeele P. (2006). Bacterial RNA and small antiviral compounds activate caspase-1 through cryopyrin/Nalp3. Nature.

[57] Sha W., Mitoma H., Hanabuchi S., Bao M., Weng L., Sugimoto N., Liu Y., Zhang Z., Zhong J., Sun B. (2014). Human NLRP3 inflammasome senses multiple types of bacterial RNAs. Proc. Natl. Acad. Sci. USA.

[58] Greaney A.J., Leppla S.H., Moayeri M. (2015). Bacterial Exotoxins and the Inflammasome. Front. Immunol..

[59] Lee M.-S., Kwon H., Lee E.-Y., Kim D.-J., Park J.-H., Tesh V.L., Oh T.-K., Kim M.H. (2016). Shiga Toxins Activate the NLRP3 Inflammasome Pathway To Promote Both Production of the Proinflammatory Cytokine Interleukin-1β and Apoptotic Cell Death. Infect. Immun..

[60] Kasper L., König A., Koenig P.-A., Gresnigt M.S., Westman J., Drummond R.A., Lionakis M.S., Groß O., Ruland J., Naglik J.R. (2018). The fungal peptide toxin Candidalysin activates the NLRP3 inflammasome and causes cytolysis in mononuclear phagocytes. Nat. Commun..

[61] Rogiers O., Frising U.C., Kucharíková S., Jabra-Rizk M.A., Loo G.v., Dijck P.V., Wullaert A. (2019). Candidalysin Crucially Contributes to Nlrp3 Inflammasome Activation by Candida albicans Hyphae. mBio.

[62] Skeldon A., Saleh M. (2011). The Inflammasomes: Molecular Effectors of Host Resistance Against Bacterial, Viral, Parasitic, and Fungal Infections. Front. Microbiol..

[63] Mathur A., Feng S., Hayward J.A., Ngo C., Fox D., Atmosukarto I.I., Price J.D., Schauer K., Märtlbauer E., Robertson A.A.B. (2019). A multicomponent toxin from Bacillus cereus incites inflammation and shapes host outcome via the NLRP3 inflammasome. Nat. Microbiol..

[64] Perregaux D., Gabel C.A. (1994). Interleukin-1 beta maturation and release in response to ATP and nigericin. Evidence that potassium depletion mediated by these agents is a necessary and common feature of their activity. J. Biol. Chem..

[65] Walev I., Klein J., Husmann M., Valeva A., Strauch S., Wirtz H., Weichel O., Bhakdi S. (2000). Potassium Regulates IL-1β Processing Via Calcium-Independent Phospholipase A2. J. Immunol..

[66] Walev I., Reske K., Palmer M., Valeva A., Bhakdi S. (1995). Potassium-inhibited processing of IL-1 beta in human monocytes. EMBO J..

[67] Muñoz-Planillo R., Kuffa P., Martínez-Colón G., Smith B.L., Rajendiran T.M., Núñez G. (2013). K^+^ efflux is the common trigger of NLRP3 inflammasome activation by bacterial toxins and particulate matter. Immunity.

[68] Pétrilli V., Papin S., Dostert C., Mayor A., Martinon F., Tschopp J. (2007). Activation of the NALP3 inflammasome is triggered by low intracellular potassium concentration. Cell Death Differ..

[69] Rühl S., Broz P. (2015). Caspase-11 activates a canonical NLRP3 inflammasome by promoting K(+) efflux. Eur. J. Immunol..

[70] Schmid-Burgk J.L., Gaidt M.M., Schmidt T., Ebert T.S., Bartok E., Hornung V. (2015). Caspase-4 mediates non-canonical activation of the NLRP3 inflammasome in human myeloid cells. Eur. J. Immunol..

[71] Yang D., He Y., Muñoz-Planillo R., Liu Q., Núñez G. (2015). Caspase-11 Requires the Pannexin-1 Channel and the Purinergic P2X7 Pore to Mediate Pyroptosis and Endotoxic Shock. Immunity.

[72] Groß C.J., Mishra R., Schneider K.S., Médard G., Wettmarshausen J., Dittlein D.C., Shi H., Gorka O., Koenig P.-A., Fromm S. (2016). K + Efflux-Independent NLRP3 Inflammasome Activation by Small Molecules Targeting Mitochondria. Immunity.

[73] Sanman L.E., Qian Y., Eisele N.A., Ng T.M., van der Linden W.A., Monack D.M., Weerapana E., Bogyo M. (2016). Disruption of glycolytic flux is a signal for inflammasome signaling and pyroptotic cell death. eLife.

[74] Meng G., Zhang F., Fuss I., Kitani A., Strober W. (2009). A Mutation in the Nlrp3 Gene Causing Inflammasome Hyperactivation Potentiates Th17 Cell-Dominant Immune Responses. Immunity.

[75] Clapham D.E. (2007). Calcium signaling. Cell.

[76] Brough D., Feuvre R.A.L., Wheeler R.D., Solovyova N., Hilfiker S., Rothwell N.J., Verkhratsky A. (2003). Ca2+ Stores and Ca2+ Entry Differentially Contribute to the Release of IL-1β and IL-1α from Murine Macrophages. J. Immunol..

[77] Feldmeyer L., Keller M., Niklaus G., Hohl D., Werner S., Beer H.-D. (2007). The inflammasome mediates UVB-induced activation and secretion of interleukin-1beta by keratinocytes. Curr. Biol. CB.

[78] Chu J., Thomas L.M., Watkins S.C., Franchi L., Núñez G., Salter R.D. (2009). Cholesterol-dependent cytolysins induce rapid release of mature IL-1β from murine macrophages in a NLRP3 inflammasome and cathepsin B-dependent manner. J. Leukoc. Biol..

[79] Murakami T., Ockinger J., Yu J., Byles V., McColl A., Hofer A.M., Horng T. (2012). Critical role for calcium mobilization in activation of the NLRP3 inflammasome. Proc. Natl. Acad. Sci. USA.

[80] Lee G.-S., Subramanian N., Kim A.I., Aksentijevich I., Goldbach-Mansky R., Sacks D.B., Germain R.N., Kastner D.L., Chae J.J. (2012). The calcium-sensing receptor regulates the NLRP3 inflammasome through Ca2+ and cAMP. Nature.

[81] Katsnelson M.A., Rucker L.G., Russo H.M., Dubyak G.R. (2015). K+ Efflux Agonists Induce NLRP3 Inflammasome Activation Independently of Ca2+ Signaling. J. Immunol..

[82] Baldwin A.G., Rivers-Auty J., Daniels M.J.D., White C.S., Schwalbe C.H., Schilling T., Hammadi H., Jaiyong P., Spencer N.G., England H. (2017). Boron-Based Inhibitors of the NLRP3 Inflammasome. Cell Chem. Biol..

[83] Compan V., Baroja-Mazo A., López-Castejón G., Gomez A.I., Martínez C.M., Angosto D., Montero M.T., Herranz A.S., Bazán E., Reimers D. (2012). Cell Volume Regulation Modulates NLRP3 Inflammasome Activation. Immunity.

[84] Zhong Z., Zhai Y., Liang S., Mori Y., Han R., Sutterwala F.S., Qiao L. (2013). TRPM2 links oxidative stress to NLRP3 inflammasome activation. Nat. Commun..

[85] Weber K., Schilling J.D. (2014). Lysosomes integrate metabolic-inflammatory cross-talk in primary macrophage inflammasome activation. J. Biol. Chem..

[86] Schorn C., Frey B., Lauber K., Janko C., Strysio M., Keppeler H., Gaipl U.S., Voll R.E., Springer E., Munoz L.E. (2011). Sodium overload and water influx activate the NALP3 inflammasome. J. Biol. Chem..

[87] Verhoef P.A., Kertesy S.B., Lundberg K., Kahlenberg J.M., Dubyak G.R. (2005). Inhibitory effects of chloride on the activation of caspase-1, IL-1beta secretion, and cytolysis by the P2X7 receptor. J. Immunol. Baltim. Md 1950.

[88] Perregaux D.G., Laliberte R.E., Gabel C.A. (1996). Human monocyte interleukin-1beta posttranslational processing. Evidence of a volume-regulated response. J. Biol. Chem..

[89] Tang T., Lang X., Xu C., Wang X., Gong T., Yang Y., Cui J., Bai L., Wang J., Jiang W. (2017). CLICs-dependent chloride efflux is an essential and proximal upstream event for NLRP3 inflammasome activation. Nat. Commun..

[90] Daniels M.J.D., Rivers-Auty J., Schilling T., Spencer N.G., Watremez W., Fasolino V., Booth S.J., White C.S., Baldwin A.G., Freeman S. (2016). Fenamate NSAIDs inhibit the NLRP3 inflammasome and protect against Alzheimer’s disease in rodent models. Nat. Commun..

[91] Domingo-Fernández R., Coll R.C., Kearney J., Breit S., O’Neill L.A.J. (2017). The intracellular chloride channel proteins CLIC1 and CLIC4 induce IL-1β transcription and activate the NLRP3 inflammasome. J. Biol. Chem..

[92] Green J.P., Yu S., Martín-Sánchez F., Pelegrin P., Lopez-Castejon G., Lawrence C.B., Brough D. (2018). Chloride regulates dynamic NLRP3-dependent ASC oligomerization and inflammasome priming. Proc. Natl. Acad. Sci. USA.

[93] Cruz C.M., Rinna A., Forman H.J., Ventura A.L.M., Persechini P.M., Ojcius D.M. (2007). ATP activates a reactive oxygen species-dependent oxidative stress response and secretion of proinflammatory cytokines in macrophages. J. Biol. Chem..

[94] van Bruggen R., Köker M.Y., Jansen M., van Houdt M., Roos D., Kuijpers T.W., van den Berg T.K. (2010). Human NLRP3 inflammasome activation is Nox1-4 independent. Blood.

[95] Ma M.W., Wang J., Dhandapani K.M., Brann D.W. (2017). NADPH Oxidase 2 Regulates NLRP3 Inflammasome Activation in the Brain after Traumatic Brain Injury. Oxid. Med. Cell. Longev..

[96] Moon J.-S., Nakahira K., Chung K.-P., DeNicola G.M., Koo M.J., Pabón M.A., Rooney K.T., Yoon J.-H., Ryter S.W., Stout-Delgado H. (2016). NOX4-dependent fatty acid oxidation promotes NLRP3 inflammasome activation in macrophages. Nat. Med..

[97] Zhou R., Yazdi A.S., Menu P., Tschopp J. (2011). A role for mitochondria in NLRP3 inflammasome activation. Nature.

[98] Nakahira K., Haspel J.A., Rathinam V.A.K., Lee S.-J., Dolinay T., Lam H.C., Englert J.A., Rabinovitch M., Cernadas M., Kim H.P. (2011). Autophagy proteins regulate innate immune responses by inhibiting the release of mitochondrial DNA mediated by the NALP3 inflammasome. Nat. Immunol..

[99] Shimada K., Crother T.R., Karlin J., Dagvadorj J., Chiba N., Chen S., Ramanujan V.K., Wolf A.J., Vergnes L., Ojcius D.M. (2012). Oxidized Mitochondrial DNA Activates the NLRP3 Inflammasome during Apoptosis. Immunity.

[100] Bauernfeind F., Bartok E., Rieger A., Franchi L., Núñez G., Hornung V. (2011). Cutting edge: Reactive oxygen species inhibitors block priming, but not activation, of the NLRP3 inflammasome. J. Immunol. Baltim. Md 1950.

[101] Park S., Juliana C., Hong S., Datta P., Hwang I., Fernandes-Alnemri T., Yu J.-W., Alnemri E.S. (2013). The mitochondrial anti-viral protein MAVS associates with NLRP3 and regulates its inflammasome activity. J. Immunol. Baltim. Md 1950.

[102] Subramanian N., Natarajan K., Clatworthy M.R., Wang Z., Germain R.N. (2013). The Adaptor MAVS Promotes NLRP3 Mitochondrial Localization and Inflammasome Activation. Cell.

[103] Ermler M.E., Traylor Z., Patel K., Schattgen S.A., Vanaja S.K., Fitzgerald K.A., Hise A.G. (2014). Rift Valley fever virus infection induces activation of the NLRP3 inflammasome. Virology.

[104] Franchi L., Eigenbrod T., Muñoz-Planillo R., Ozkurede U., Kim Y.-G., Chakrabarti A., Gale M., Silverman R.H., Colonna M., Akira S. (2014). Cytosolic Double-Stranded RNA Activates the NLRP3 Inflammasome via MAVS-Induced Membrane Permeabilization and K+ Efflux. J. Immunol..

[105] Guan K., Wei C., Zheng Z., Song T., Wu F., Zhang Y., Cao Y., Ma S., Chen W., Xu Q. (2015). MAVS Promotes Inflammasome Activation by Targeting ASC for K63-Linked Ubiquitination via the E3 Ligase TRAF3. J. Immunol..

[106] Ichinohe T., Yamazaki T., Koshiba T., Yanagi Y. (2013). Mitochondrial protein mitofusin 2 is required for NLRP3 inflammasome activation after RNA virus infection. Proc. Natl. Acad. Sci. USA.

[107] Iyer S.S., He Q., Janczy J.R., Elliott E.I., Zhong Z., Olivier A.K., Sadler J.J., Knepper-Adrian V., Han R., Qiao L. (2013). Mitochondrial cardiolipin is required for Nlrp3 inflammasome activation. Immunity.

[108] Elliott E.I., Miller A.N., Banoth B., Iyer S.S., Stotland A., Weiss J.P., Gottlieb R.A., Sutterwala F.S., Cassel S.L. (2018). Cutting Edge: Mitochondrial Assembly of the NLRP3 Inflammasome Complex Is Initiated at Priming. J. Immunol..

[109] Misawa T., Takahama M., Kozaki T., Lee H., Zou J., Saitoh T., Akira S. (2013). Microtubule-driven spatial arrangement of mitochondria promotes activation of the NLRP3 inflammasome. Nat. Immunol..

[110] Wang Y., Yang C., Mao K., Chen S., Meng G., Sun B. (2013). Cellular localization of NLRP3 inflammasome. Protein Cell.

[111] Chen J., Chen Z.J. (2018). PtdIns4P on dispersed trans -Golgi network mediates NLRP3 inflammasome activation. Nature.

[112] Zhang Z., Meszaros G., He W., Xu Y., Magliarelli H.d.F., Mailly L., Mihlan M., Liu Y., Gámez M.P., Goginashvili A. (2017). Protein kinase D at the Golgi controls NLRP3 inflammasome activation. J. Exp. Med..

[113] Duewell P., Kono H., Rayner K.J., Sirois C.M., Vladimer G., Bauernfeind F.G., Abela G.S., Franchi L., Nuñez G., Schnurr M. (2010). NLRP3 inflammasomes are required for atherogenesis and activated by cholesterol crystals. Nature.

[114] Cassel S.L., Eisenbarth S.C., Iyer S.S., Sadler J.J., Colegio O.R., Tephly L.A., Carter A.B., Rothman P.B., Flavell R.A., Sutterwala F.S. (2008). The Nalp3 inflammasome is essential for the development of silicosis. Proc. Natl. Acad. Sci. USA.

[115] Halle A., Hornung V., Petzold G.C., Stewart C.R., Monks B.G., Reinheckel T., Fitzgerald K.A., Latz E., Moore K.J., Golenbock D.T. (2008). The NALP3 inflammasome is involved in the innate immune response to amyloid-β. Nat. Immunol..

[116] Kool M., Pétrilli V., De Smedt T., Rolaz A., Hammad H., van Nimwegen M., Bergen I.M., Castillo R., Lambrecht B.N., Tschopp J. (2008). Cutting edge: Alum adjuvant stimulates inflammatory dendritic cells through activation of the NALP3 inflammasome. J. Immunol. Baltim. Md 1950.

[117] Codolo G., Plotegher N., Pozzobon T., Brucale M., Tessari I., Bubacco L., de Bernard M. (2013). Triggering of Inflammasome by Aggregated α–Synuclein, an Inflammatory Response in Synucleinopathies. PLoS ONE.

[118] Dostert C., Guarda G., Romero J.F., Menu P., Gross O., Tardivel A., Suva M.-L., Stehle J.-C., Kopf M., Stamenkovic I. (2009). Malarial Hemozoin Is a Nalp3 Inflammasome Activating Danger Signal. PLoS ONE.

[119] Orlowski G.M., Colbert J.D., Sharma S., Bogyo M., Robertson S.A., Rock K.L. (2015). Multiple Cathepsins Promote Pro-IL-1β Synthesis and NLRP3-Mediated IL-1β Activation. J. Immunol. Baltim. Md 1950.

[120] Barlan A.U., Griffin T.M., McGuire K.A., Wiethoff C.M. (2011). Adenovirus membrane penetration activates the NLRP3 inflammasome. J. Virol..

[121] Hagar J.A., Powell D.A., Aachoui Y., Ernst R.K., Miao E.A. (2013). Cytoplasmic LPS Activates Caspase-11: Implications in TLR4-Independent Endotoxic Shock. Science.

[122] Kayagaki N., Wong M.T., Stowe I.B., Ramani S.R., Gonzalez L.C., Akashi-Takamura S., Miyake K., Zhang J., Lee W.P., Muszyński A. (2013). Noncanonical Inflammasome Activation by Intracellular LPS Independent of TLR4. Science.

[123] Balakrishnan A., Karki R., Berwin B., Yamamoto M., Kanneganti T.-D. (2018). Guanylate binding proteins facilitate caspase-11-dependent pyroptosis in response to type 3 secretion system-negative Pseudomonas aeruginosa. Cell Death Discov..

[124] Kayagaki N., Warming S., Lamkanfi M., Walle L.V., Louie S., Dong J., Newton K., Qu Y., Liu J., Heldens S. (2011). Non-canonical inflammasome activation targets caspase-11. Nature.

[125] Baker P.J., Boucher D., Bierschenk D., Tebartz C., Whitney P.G., D’Silva D.B., Tanzer M.C., Monteleone M., Robertson A.A.B., Cooper M.A. (2015). NLRP3 inflammasome activation downstream of cytoplasmic LPS recognition by both caspase-4 and caspase-5. Eur. J. Immunol..

[126] Shi J., Zhao Y., Wang Y., Gao W., Ding J., Li P., Hu L., Shao F. (2014). Inflammatory caspases are innate immune receptors for intracellular LPS. Nature.

[127] Broz P., Ruby T., Belhocine K., Bouley D.M., Kayagaki N., Dixit V.M., Monack D.M. (2012). Caspase-11 increases susceptibility to *Salmonella* infection in the absence of caspase-1. Nature.

[128] Pelegrin P., Surprenant A. (2006). Pannexin-1 mediates large pore formation and interleukin-1beta release by the ATP-gated P2X7 receptor. EMBO J..

[129] Piccini A., Carta S., Tassi S., Lasiglié D., Fossati G., Rubartelli A. (2008). ATP is released by monocytes stimulated with pathogen-sensing receptor ligands and induces IL-1β and IL-18 secretion in an autocrine way. Proc. Natl. Acad. Sci. USA.

[130] Alves L.A., de Melo Reis R.A., de Souza C.A.M., de Freitas M.S., Teixeira P.C.N., Neto Moreira Ferreira D., Xavier R.F. (2014). The P2X7 receptor: Shifting from a low- to a high-conductance channel—An enigmatic phenomenon?. Biochim. Biophys. Acta BBA-Biomembr..

[131] Chu L.H., Indramohan M., Ratsimandresy R.A., Gangopadhyay A., Morris E.P., Monack D.M., Dorfleutner A., Stehlik C. (2018). The oxidized phospholipid oxPAPC protects from septic shock by targeting the non-canonical inflammasome in macrophages. Nat. Commun..

[132] Man S.M., Karki R., Sasai M., Place D.E., Kesavardhana S., Temirov J., Frase S., Zhu Q., Malireddi R.K.S., Kuriakose T. (2016). IRGB10 Liberates Bacterial Ligands for Sensing by the AIM2 and Caspase-11-NLRP3 Inflammasomes. Cell.

[133] Meunier E., Dick M.S., Dreier R.F., Schürmann N., Kenzelmann Broz D., Warming S., Roose-Girma M., Bumann D., Kayagaki N., Takeda K. (2014). Caspase-11 activation requires lysis of pathogen-containing vacuoles by IFN-induced GTPases. Nature.

[134] Netea M.G., Nold-Petry C.A., Nold M.F., Joosten L.A.B., Opitz B., van der Meer J.H.M., van de Veerdonk F.L., Ferwerda G., Heinhuis B., Devesa I. (2009). Differential requirement for the activation of the inflammasome for processing and release of IL-1beta in monocytes and macrophages. Blood.

[135] Gaidt M.M., Ebert T.S., Chauhan D., Schmidt T., Schmid-Burgk J.L., Rapino F., Robertson A.A.B., Cooper M.A., Graf T., Hornung V. (2016). Human Monocytes Engage an Alternative Inflammasome Pathway. Immunity.

[136] He Y., Franchi L., Núñez G. (2013). TLR Agonists Stimulate Nlrp3-Dependent IL-1β Production Independently of the Purinergic P2X7 Receptor in Dendritic Cells and In Vivo. J. Immunol..

[137] Yang J., Liu Z., Xiao T.S. (2017). Post-translational regulation of inflammasomes. Cell. Mol. Immunol..

[138] Han S., Lear T.B., Jerome J.A., Rajbhandari S., Snavely C.A., Gulick D.L., Gibson K.F., Zou C., Chen B.B., Mallampalli R.K. (2015). Lipopolysaccharide Primes the NALP3 Inflammasome by Inhibiting Its Ubiquitination and Degradation Mediated by the SCFFBXL2 E3 Ligase. J. Biol. Chem..

[139] Yan Y., Jiang W., Liu L., Wang X., Ding C., Tian Z., Zhou R. (2015). Dopamine Controls Systemic Inflammation through Inhibition of NLRP3 Inflammasome. Cell.

[140] Song H., Liu B., Huai W., Yu Z., Wang W., Zhao J., Han L., Jiang G., Zhang L., Gao C. (2016). The E3 ubiquitin ligase TRIM31 attenuates NLRP3 inflammasome activation by promoting proteasomal degradation of NLRP3. Nat. Commun..

[141] Kawashima A., Karasawa T., Tago K., Kimura H., Kamata R., Usui-Kawanishi F., Watanabe S., Ohta S., Funakoshi-Tago M., Yanagisawa K. (2017). ARIH2 Ubiquitinates NLRP3 and Negatively Regulates NLRP3 Inflammasome Activation in Macrophages. J. Immunol..

[142] Humphries F., Bergin R., Jackson R., Delagic N., Wang B., Yang S., Dubois A.V., Ingram R.J., Moynagh P.N. (2018). The E3 ubiquitin ligase Pellino2 mediates priming of the NLRP3 inflammasome. Nat. Commun..

[143] Palazón-Riquelme P., Worboys J.D., Green J., Valera A., Martín-Sánchez F., Pellegrini C., Brough D., López-Castejón G. (2018). USP7 and USP47 deubiquitinases regulate NLRP3 inflammasome activation. EMBO Rep..

[144] Sandall C.F., MacDonald J.A. (2019). Effects of phosphorylation on the NLRP3 inflammasome. Arch. Biochem. Biophys..

[145] Mortimer L., Moreau F., MacDonald J.A., Chadee K. (2016). NLRP3 inflammasome inhibition is disrupted in a group of auto-inflammatory disease CAPS mutations. Nat. Immunol..

[146] Guo C., Xie S., Chi Z., Zhang J., Liu Y., Zhang L., Zheng M., Zhang X., Xia D., Ke Y. (2016). Bile Acids Control Inflammation and Metabolic Disorder through Inhibition of NLRP3 Inflammasome. Immunity.

[147] Spalinger M.R., Kasper S., Gottier C., Lang S., Atrott K., Vavricka S.R., Scharl S., Gutte P.M., Grütter M.G., Beer H.-D. (2016). NLRP3 tyrosine phosphorylation is controlled by protein tyrosine phosphatase PTPN22. J. Clin. Investig..

[148] Stutz A., Kolbe C.-C., Stahl R., Horvath G.L., Franklin B.S., van Ray O., Brinkschulte R., Geyer M., Meissner F., Latz E. (2017). NLRP3 inflammasome assembly is regulated by phosphorylation of the pyrin domain. J. Exp. Med..

[149] Hernandez-Cuellar E., Tsuchiya K., Hara H., Fang R., Sakai S., Kawamura I., Akira S., Mitsuyama M. (2012). Cutting edge: Nitric oxide inhibits the NLRP3 inflammasome. J. Immunol. Baltim. Md 1950.

[150] Mao K., Chen S., Chen M., Ma Y., Wang Y., Huang B., He Z., Zeng Y., Hu Y., Sun S. (2013). Nitric oxide suppresses NLRP3 inflammasome activation and protects against LPS-induced septic shock. Cell Res..

[151] Mishra B.B., Rathinam V.A.K., Martens G.W., Martinot A.J., Kornfeld H., Fitzgerald K.A., Sassetti C.M. (2013). Nitric oxide controls the immunopathology of tuberculosis by inhibiting NLRP3 inflammasome-dependent processing of IL-1β. Nat. Immunol..

[152] Barry R., John S.W., Liccardi G., Tenev T., Jaco I., Chen C.-H., Choi J., Kasperkiewicz P., Fernandes-Alnemri T., Alnemri E. (2018). SUMO-mediated regulation of NLRP3 modulates inflammasome activity. Nat. Commun..

[153] Bose S., Segovia J.A., Somarajan S.R., Chang T.-H., Kannan T.R., Baseman J.B. (2014). ADP-Ribosylation of NLRP3 by Mycoplasma pneumoniae CARDS Toxin Regulates Inflammasome Activity. mBio.

[154] Mayor A., Martinon F., De Smedt T., Pétrilli V., Tschopp J. (2007). A crucial function of SGT1 and HSP90 in inflammasome activity links mammalian and plant innate immune responses. Nat. Immunol..

[155] Piippo N., Korhonen E., Hytti M., Skottman H., Kinnunen K., Josifovska N., Petrovski G., Kaarniranta K., Kauppinen A. (2018). Hsp90 inhibition as a means to inhibit activation of the NLRP3 inflammasome. Sci. Rep..

[156] Li F., Song X., Su G., Wang Y., Wang Z., Qing S., Jia J., Wang Y., Huang L., Zheng K. (2019). AT-533, a Hsp90 inhibitor, attenuates HSV-1-induced inflammation. Biochem. Pharmacol..

[157] Zuo Y., Wang J., Liao F., Yan X., Li J., Huang L., Liu F. (2018). Inhibition of Heat Shock Protein 90 by 17-AAG Reduces Inflammation via P2X7 Receptor/NLRP3 Inflammasome Pathway and Increases Neurogenesis After Subarachnoid Hemorrhage in Mice. Front. Mol. Neurosci..

[158] Zhou R., Tardivel A., Thorens B., Choi I., Tschopp J. (2010). Thioredoxin-interacting protein links oxidative stress to inflammasome activation. Nat. Immunol..

[159] Masters S.L., Dunne A., Subramanian S.L., Hull R.L., Tannahill G.M., Sharp F.A., Becker C., Franchi L., Yoshihara E., Chen Z. (2010). Activation of the NLRP3 inflammasome by islet amyloid polypeptide provides a mechanism for enhanced IL-1β in type 2 diabetes. Nat. Immunol..

[160] Shenoy A.R., Wellington D.A., Kumar P., Kassa H., Booth C.J., Cresswell P., MacMicking J.D. (2012). GBP5 Promotes NLRP3 Inflammasome Assembly and Immunity in Mammals. Science.

[161] Man S.M., Karki R., Malireddi R.K.S., Neale G., Vogel P., Yamamoto M., Lamkanfi M., Kanneganti T.-D. (2015). The transcription factor IRF1 and guanylate-binding proteins target AIM2 inflammasome activation by Francisella infection. Nat. Immunol..

[162] Meunier E., Wallet P., Dreier R.F., Costanzo S., Anton L., Rühl S., Dussurgey S., Dick M.S., Kistner A., Rigard M. (2015). Guanylate-binding proteins promote activation of the AIM2 inflammasome during infection with Francisella novicida. Nat. Immunol..

[163] Lu B., Nakamura T., Inouye K., Li J., Tang Y., Lundbäck P., Valdes-Ferrer S.I., Olofsson P.S., Kalb T., Roth J. (2012). Novel role of PKR in inflammasome activation and HMGB1 release. Nature.

[164] Yoshida K., Okamura H., Hiroshima Y., Abe K., Kido J.-I., Shinohara Y., Ozaki K. (2017). PKR induces the expression of NLRP3 by regulating the NF-κB pathway in Porphyromonas gingivalis-infected osteoblasts. Exp. Cell Res..

[165] He Y., Franchi L., Núñez G. (2013). The protein kinase PKR is critical for LPS-induced iNOS production but dispensable for inflammasome activation in macrophages. Eur. J. Immunol..

[166] Li X., Thome S., Ma X., Amrute-Nayak M., Finigan A., Kitt L., Masters L., James J.R., Shi Y., Meng G. (2017). MARK4 regulates NLRP3 positioning and inflammasome activation through a microtubule-dependent mechanism. Nat. Commun..

[167] Lang T., Lee J.P.W., Elgass K., Pinar A.A., Tate M.D., Aitken E.H., Fan H., Creed S.J., Deen N.S., Traore D.A.K. (2018). Macrophage migration inhibitory factor is required for NLRP3 inflammasome activation. Nat. Commun..

[168] He Y., Zeng M.Y., Yang D., Motro B., Núñez G. (2016). NEK7 is an essential mediator of NLRP3 activation downstream of potassium efflux. Nature.

[169] Schmid-Burgk J.L., Chauhan D., Schmidt T., Ebert T.S., Reinhardt J., Endl E., Hornung V. (2016). A Genome-wide CRISPR (Clustered Regularly Interspaced Short Palindromic Repeats) Screen Identifies NEK7 as an Essential Component of NLRP3 Inflammasome Activation. J. Biol. Chem..

[170] Shi H., Wang Y., Li X., Zhan X., Tang M., Fina M., Su L., Pratt D., Bu C.H., Hildebrand S. (2016). NLRP3 activation and mitosis are mutually exclusive events coordinated by NEK7, a new inflammasome component. Nat. Immunol..

[171] Salem H., Rachmin I., Yissachar N., Cohen S., Amiel A., Haffner R., Lavi L., Motro B. (2010). Nek7 kinase targeting leads to early mortality, cytokinesis disturbance and polyploidy. Oncogene.

